# Case report: Mucous membrane pemphigoid with complicated autoantibody profile indicating the necessity of comprehensive diagnostic methods and the contribution of IgA autoantibodies

**DOI:** 10.3389/fimmu.2023.1149119

**Published:** 2023-03-09

**Authors:** Weijun Liu, Huicheng Li, Yun Jin, Lifang Cheng, Luhuai Shi, Yangmin Gao, Zhijun Zhou, Suying Feng, Hua Qian, Takashi Hashimoto, Xiaoguang Li

**Affiliations:** ^1^ Dermatology Hospital of Jiangxi Province, Jiangxi Provincial Clinical Research Center for Skin Diseases, Candidate Branch of National Clinical Research Center for Skin Diseases, Dermatology Institute of Jiangxi Province, The Affiliated Dermatology Hospital of Nanchang University, Nanchang, China; ^2^ Key Laboratory of Modern Preparation of Traditional Chinese Medicine, Ministry of Education, Jiangxi University of Traditional Chinese Medicine, Nanchang, China; ^3^ Institute of Dermatology, Chinese Academy of Medical Sciences and Peking Union Medical College, Nanjing, China; ^4^ Department of Laboratory Medicine, Chronic Disease Research Center, Medical College, Dalian University, Dalian, China; ^5^ Department of Dermatology, Osaka Metropolitan University Graduate School of Medicine, Osaka, Japan

**Keywords:** mucous membrane pemphigoid, IgA autoantibody, lesions on the extremities, bronchiolitis obliterans, integrin α6β4, laminin 332, BP180

## Abstract

Mucous membrane pemphigoid (MMP) is a type of subepithelial autoimmune bullous disease, affecting various mucosae, occasionally with skin lesions. Both diagnosis and treatment of MMP are difficult. Although multiple autoantigens have been identified for MMP, the pathogenesis of MMP is still unclear. In this study, we presented a female MMP case with extensive oral mucosal lesions and skin lesions, particularly on the extremities. IgG and IgA autoantibodies against multiple autoantigens including BP180, laminin 332, integrinα6β4 and desmoglein 3, and IgM autoantibodies against BP180 were identified during the disease course. Compared with IgG autoantibodies, the levels of IgA autoantibodies against various autoantigens decreased more significantly with improvement of clinical features after the initiation of treatments. Our findings indicated the importance of comprehensive autoantibody screening for different immunoglobulin types and autoantigens at multiple time points for the precise diagnosis of various autoimmune bullous diseases, and the significant involvement of IgA autoantibodies into the pathogenesis of MMP.

## Introduction

Mucous membrane pemphigoid (MMP) is a subgroup of chronic, inflammatory, subepithelial autoimmune bullous disease (AIBD), mainly affecting various mucosal membranes, particularly oral and ocular mucosae, with or without skin lesions (1–3). Identified MMP-related autoantigens include BP180, BP230, laminin (LM) 332, type VII collagen, integrin (ITG) α6β4 and LMγ1 (1, 4, 5), among which autoantibodies against BP180 and LM332 have been most frequently reported (1–3, 6–8). MMP-related autoantibodies are mainly IgG type and less frequently IgA type, and MMP cases occasionally showed multiple autoantibodies (1–4). Our group has continuously focused on the research of MMP cases and attempted to find autoantibodies relevant for the diagnosis and pathogenesis by analyzing the clinical features and the autoantibody profiles of each MMP case during the disease course.

In this study, we presented a unique MMP case with autoantibodies of different immunoglobulin types against multiple autoantigens, which emphasized the diagnostic and pathogenenic values of IgA autoantibodies in MMP. In addition, the findings in the present case suggested the potential relationship between predominant lesions on the extremities and the presence of anti-BP180 autoantibodies, which we hypothesized in our previous study (9).

## Case description

**Figure 1:**
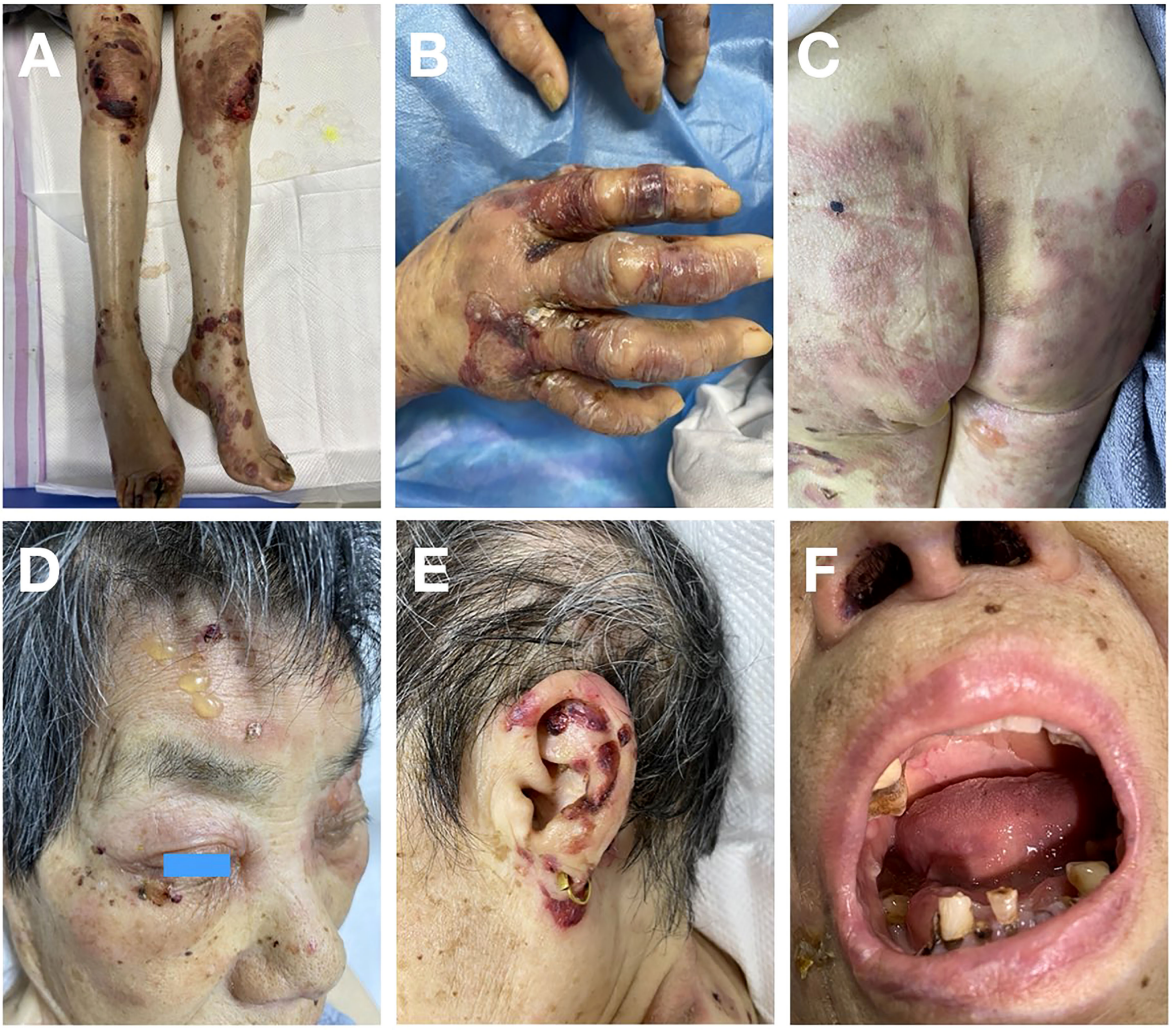
Clinical features of this patient. **(A, B)** Erythema, blisters and bloody crusts on the knees and lower limbs **(A)** and on the hands **(B)**. **(C)** Erythema and blisters on the buttocks and thighs. **(D)** Blisters on the face and bloody crusts around the eye. **(E)** Bloody crusts around the ear. **(F)** Sever mucosal lesions on the lips and in the oral cavity.

**Figure 2:**
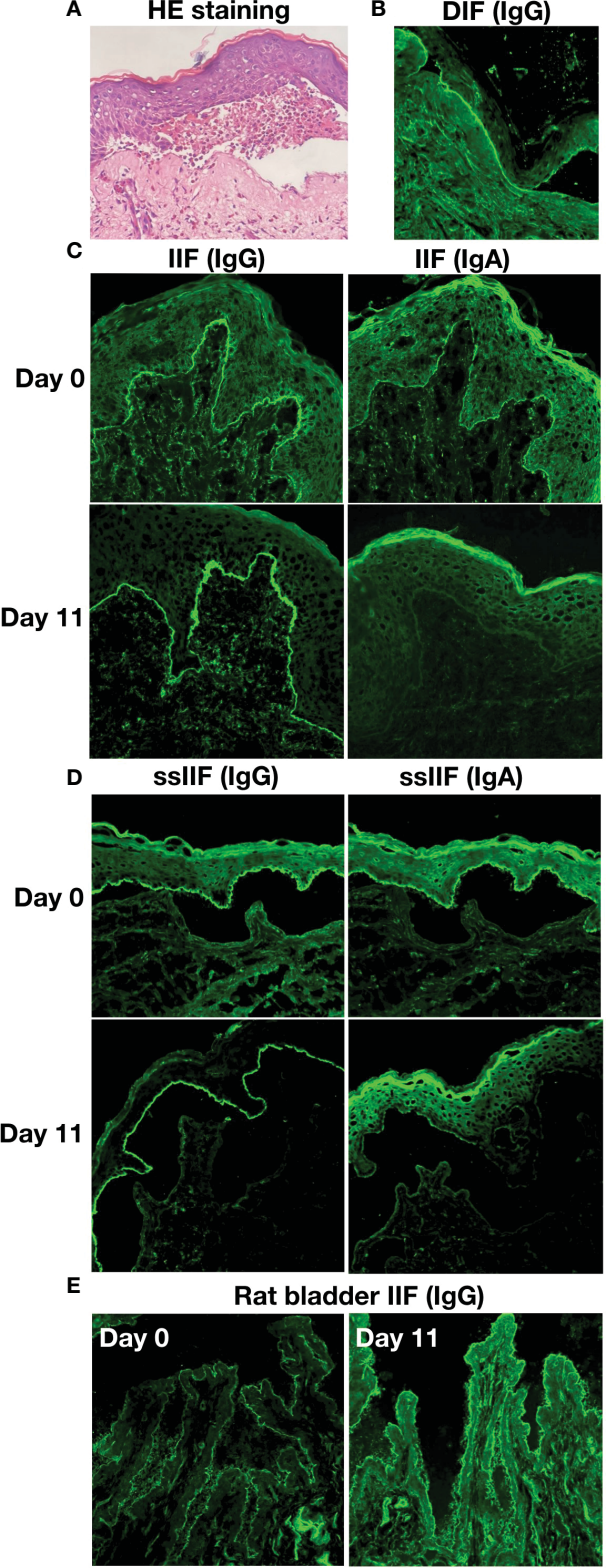
The results of histopathological and immunofluorescence tests of this patient. **(A)** Histopathological features of biopsy from lesioned skin on the left forearm (HE staining, original magnification, x200). **(B)** Direct immunofluorescence (DIF) for IgG. **(C)** Indirect immunofluorescence with normal human skin (IIF) for IgG and IgA antibodies on Days 0 and 11. **(D)** IIF using 1 M NaCl-split normal human skin (ssIIF) for IgG and IgA antibodies on Days 0 and 11. **(E)** IIF using rat bladder tissue (Rat bladder IIF) for IgG antibodies on Days 0 and 11.

An 80-year-old female presented with scattered erythema, blisters, erosions and bloody crusts with itch on the head, trunk and limbs (**Figures 1A–E**), accompanied with extensive oral mucosal lesions (**Figure 1F**). Histopathology of biopsy from lesional skin on the left forearm showed subepidermal blister and infiltration of lymphocytes and eosinophils (**Figure 2A**). Direct immunofluorescence showed only linear basement membrane zone (BMZ) deposition of IgG (**Figure 2B**), but not of IgA, IgM and C3 (data not shown), without depositions to keratinocyte cell surfaces.

By indirect immunofluorescence (IIF) using normal human skin, the patient serum taken at Day 0 showed IgG and IgA anti-BMZ antibodies, without antibodies to keratinocyte cell surfaces (**Figure 2C**), while the serum of Day 11 showed only IgG anti-BMZ antibodies (**Figure 2C**). By IIF using 1M NaCl-split normal human skin (ssIIF), the patient serum of Day 0 showed IgG and IgA antibodies reactive with the epidermal side of the split skin (**Figure 2D**), while the serum of Day 11 showed strong IgG and weaker IgA reactivities to the epidermal side of the split skin (**Figure 2D**). IIF using rat bladder tissue showed IgG staining on transitional epithelia weakly for the serum of Day 0 and strongly for the serum of Day 11 (**Figure 2E**).

Clinical features and treatments during the disease course are summarized in **Table 1**.

**Table 1:** Clinical features and treatment during the disease course.

Days	Clinical features	Treatment
Before visit to our hospital	Erythema and blisters with itch on the head, trunk and limbs, with oral mucosal lesions for 20 days	No treatments
Day 0	Erythema, blisters, erosions and bloody crusts with itch on the head, trunk and limbs	Prednisone 15 mg/day, minocycline hydrochloride 100 mg/day, thalidomide 50 mg/da
Day 11	There were no new erythema or blisters or erythema, and no itching. New oral mucosal lesions appeared.	Prednisone 15 mg/day, minocycline hydrochloride 100 mg/day, thalidomide 50 mg/day
Day 57	There were no new lesions and no itching. Dyspnea developed	Prednisone 10 mg/day, minocycline hydrochloride 100 mg/day, thalidomide 50 mg/day
Day 105	There were no new lesions, and no itching. Diagnosis of bronchiolitis obliterans was made.	Prednisone 5 mg/day, minocycline hydrochloride 100 mg/day, thalidomide 50 mg/day

**Figure 3:**
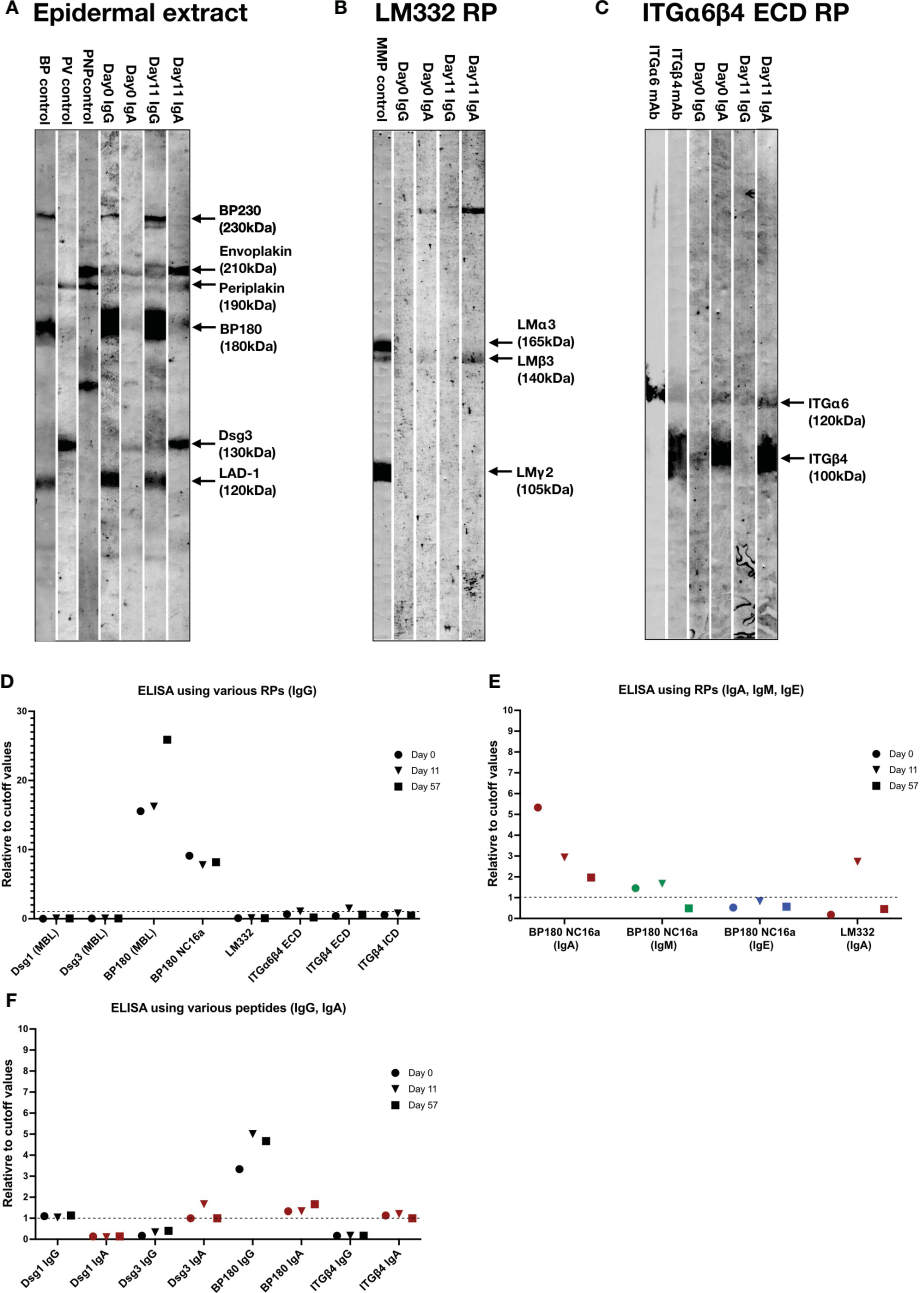
The results of immunoblotting tests and ELISAs of this patient. **(A–C)** Immunoblotting using normal human epidermal extract **(A)**, recombinant proteins (RP) of laminin (LM) 332 **(B)** and RP of extracellular domain (ECD) of integrin (ITG) α6β4 **(C)**. IgG antibodies of bullous pemphigoid (BP) control serum were positive for BP230, BP180 and LAD-1, IgG antibodies of pemphigus vulgaris (PV) control serum were positive for desmoglein (Dsg) 1 and Dsg3, IgG antibodies of paraneoplastic pemphigus (PNP) control serum were positive for envoplakin and periplakin. IgG antibodies of anti-LM332-type mucous membrane pemphigoid (MMP) control serum were positive for three subunits of LM332. mAb, monoclonal antibody. The RPs of LM332 and ITGα6β4 ECD were purchased from Biolamina (Sundbyberg, Sweden) and R&D systems (Minneapolis, MN, USA), respectively. **(D)** ELISAs using various RPs, including three commercially available ELSIA kits (MBL, Tokyo, Japan) and other in-house ELISAs for detection of IgG autoantibodies against corresponding autoantigens. **(E)** ELISAs using BP180 NC16a-domain and LM332 RPs for detection of IgA, IgM or IgE autoantibodies. **(F)** ELISAs using peptides of indicated autoantigens for detection of corresponding IgG and IgA autoantibodies. Dot lines indicate the cutoff values.

We next performed various immunoblotting tests and ELISAs for this patient sera taken at Days 0, 11 and/or 57 (**Table S1**). By immunoblotting of normal human epidermal extract, the sera of Days 0 and 11 showed IgG autoantibodies against BP230, BP180 and LAD-1, and IgA autoantibodies against envoplakin, periplakin and desmoglein (Dsg) 3, with relatively stronger IgA reactivities for the serum of Day 11(**Figure 3A**). By immunoblotting of LM332 recombinant protein (RP), the serum of Day 11, but not of Day 0, showed IgA autoantibodies against LMβ3 (**Figure 3B**). By immunoblotting of RP of extracellular domain (ECD) of ITGα6β4, the sera of both Days 0 and 11 showed IgA autoantibodies against ITGα6 and ITGβ4 (**Figure 3C**).

By ELISAs using various RPs, IgG autoantibodies against BP180 NC16a-domain were positive for all the sera of Days 0, 11 and 57, IgG autoantibodies against RPs of ECDs of ITGα6β4 and ITGβ4 were weakly positive for only the serum of Day 11, while all the sera were negative for IgG autoantibodies against Dsg1, Dsg3 and LM332 (**Figure 3D**).

By ELISAs of BP180 NC16a-domain RP, IgA autoantibodies were positive at Days 0, 11 and 57 with decreasing levels with time, and IgM autoantibodies were positive at Days 0 and 11, but negative at Day 57, while IgE autoantibodies were continuously negative (**Figure 3E**). By ELISA of LM332 RP, IgA autoantibodies against LM332 was positive at Day 11, but negative at Days 0 and 57 (**Figure 3E**).

By ELISA of peptides of various autoantigens, IgG autoantibodies against Dsg1 and BP180, but not Dsg3 and ITGβ4, were positive at Days 0, 11 and 57, and IgA autoantibodies against Dsg3, BP180 and ITGβ4, but not Dsg1, were positive at Days 0, 11 and 57, although their levels changed with time (**Figure 3F**).

The sera of this patient showed negative results in other tests, which are routinely performed in our laboratory for detection of other known autoantibodies in AIBDs.

Based on the clinical, histopathological features and immunological findings, this patient was diagnosed as MMP with IgG autoantibodies against BP230, BP180, LAD-1, Dsg1, ITGα6β4 and IgA autoantibodies against BP180, LM332, ITGα6β4, Dsg3, envoplakin and periplakin.

The treatment with prednisone 15mg/day was initiated with other drugs described in **Table 1**. The oral mucosal lesions were most severe at Day 11, and then improved with therapy. The HSV DNA was detected in the oral secretions at Day 11. The dose of prednisone tapered to 10 mg/day at Day 57, when no new mucocutaneous lesions developed (**Table 1**). In addition, the patient was developing dyspnea during the disease course and diagnosis of bronchiolitis obliterans was made at Day 105 (**Table 1**).

## Discussion

This case showed very complicated immunological profile of IgG and IgA autoantibodies to multiple autoantigens during the disease course, although this case might be simply diagnosed as bullous pemphigoid or anti-BP180-type MMP, if the comprehensive autoantibody screening was not performed. The results in this study emphasized the importance of comprehensive autoantibody screening with different immunoglobulin types for multiple time points (4, 10).

Compared with Day 0, the serum of Day 11 showed relatively lower IgA reactivities in IIF and ssIIF, which was consistent with the levels of IgA anti-BP180 NC16a-domain autoantibodies detected by ELISA. However, compared with Day 0, the serum of Day 11 had increased levels of IgA autoantibodies against BP180, Dsg3, LM332 and ITGα6β4 detected by immunoblotting and ELISAs of RPs, and increased levels of IgA autoantibodies against Dsg3, BP180 and ITGβ4 detected by ELISAs of peptides, which implied that those increased IgA autoantibodies might be responsible for the severer oral mucosal lesions at Day 11. Compared with Day 11, the serum of Day 57 showed decreased levels of IgA autoantibodies against Dsg3 and ITGβ4 detected by ELISAs of peptides. Although various IgA autoantibodies in the present case showed distinct pattern of intensities, their levels were finally decreased with accordance of improvement of clinical features after the treatments. In contrast, the levels of IgG against multiple autoantigens showed no significant decreases with therapy. These results implied that IgA autoantibodies in this patient might be the major pathogenic autoantibodies.

The complicated dynamics of various autoantibodies appearance in the disease course of this patient could be justified by epitope spreading phenomena (11).

This case showed positive IgG signals on transitional epithelia in rat bladder IIF, demonstrated autoantibodies against envoplakin and periplakin in immunoblotting of normal human epidermal extract, and was diagnosed with bronchiolitis obliterans, which suggested the possible diagnosis of paraneoplastic pemphigus (PNP) (9). However, this case showed prominent reactivity with BMZ in both direct and indirect immunofluorescence tests, strongly reacted with various MMP-related autoantigens, and showed no tumors. Therefore, we finally diagnosed this case as MMP, rather than PNP (sine neoplasia). Various findings in this case strongly suggested the possible relationship between MMP and PNP.

We have previously reported that skin lesions on the extremities were seen significantly more frequently in PNP patients with positive anti-BP180 autoantibodies (9). The clinical features and autoantibody profile of the present case also support this conclusion.

In addition, autoantibodies against envoplakin and periplakin were important diagnostic markers for PNP, and a considerable number of PNP patients would later develop bronchiolitis obliterans (9). Therefore, we speculate that autoantibodies against envoplakin and periplakin might also be predictive markers for bronchiolitis obliterans. In the present case, autoantibodies against envoplakin and periplakin were detected in the patient serum of Day 11, and bronchiolitis obliterans was finally confirmed at Day 105, which suggested the possible relationship between anti-envoplakin/periplakin autoantibodies and bronchiolitis obliterans.

We recently investigated the correlation of HSA infection and pemphigus vulgaris (PV), and found that the significantly elevated prevalence of HSV infection in PV patients might mainly be caused by ruptured barrier of oral mucosa (12). HSV infection in MMP patients was rarely reported (13), and its potential participation mechanism in the present MMP case is still unclear.

In the present MMP case, prednisone combined with minocycline hydrochloride, and thalidomide had a good therapeutic effect.

In summary, we reported a MMP case with complicated IgG and IgA autoantibody profile against against multiple autoantigens. The results in the present report will benefit for future pathogenesis studies of MMP, particularly on IgA autoantibodies and involvement of multiple autoantibodies.

## Data availability statement

The original contributions presented in the study are included in the article/**Supplementary Material**. Further inquiries can be directed to the corresponding authors.

## Ethics statement

This study was performed according to Declaration of Helsinki and approved by ethics committee of Dalian University. The patients/participants provided their written informed consent to participate in this study. Written informed consent was obtained from the individual(s) for the publication of any potentially identifiable images or data included in this article.

## Author contributions

WL, HQ, TH and XL had full access to all of the data in the study and took responsibility for the integrity of the data and the accuracy of the data analysis. HQ, TH and XL conceived and designed the project. WL, HL, YJ, LC, LS, YG, ZZ, SF, HQ, TH, XL collected case information and laboratory data, and analyzed the data. All authors contributed to the article and approved the submitted version.
