# Supplementary material for: Case report: Mucous membrane pemphigoid with complicated autoantibody profile indicating the necessity of comprehensive diagnostic methods and the contribution of IgA autoantibodies
Source: Front Immunol. 2023 Mar 9;14:1149119. doi: 10.3389/fimmu.2023.1149119 (PMC10033602; doi:10.3389/fimmu.2023.1149119)
Supplement: Supplementary file 1 [file Table_1.docx]

**Supplementary data**

**Table S1. The results of immunoblotting and ELISA tests for the sera of this patient collected at different time points**

| **Methods** |  | **Day 0** | **Day 11** | **Day 57** |
| --- | --- | --- | --- | --- |
| IB of epidermal extract | |  |  |  |
|  | BP230 IgG | (+) | (+) | / |
|  | BP230 IgA | (-) | (-) | / |
|  | BP180 IgG | (+) | (+) | / |
|  | BP180 IgA | (-) | (-) | / |
|  | LAD-1 IgG | (+) | (+) | / |
|  | LAD-1 IgA | (-) | (-) | / |
|  | Envoplakin IgG | (-) | (-) | / |
|  | Envoplakin IgA | (+) | (+) | / |
|  | Periplakin IgG | (-) | (-) | / |
|  | Periplakin IgA | (+) | (+) | / |
|  | Dsg3 IgG | (-) | (-) | / |
|  | Dsg3 IgA | (+) | (+) | / |
|  |  |  |  |  |
| IB of LM332 RP | |  |  |  |
|  | LMα3 IgG | (-) | (-) | / |
|  | LMα3 IgA | (-) | (-) | / |
|  | LMβ3 IgG | (-) | (-) | / |
|  | LMβ3 IgA | (-) | (+) | / |
|  | LMγ2 IgG | (-) | (-) | / |
|  | LMγ2 IgA | (-) | (-) | / |
|  |  |  |  |  |
| IB of ITGα6β4 ECD RP | |  |  |  |
|  | ITGα6 IgG | (-) | (-) | / |
|  | ITGα6 IgA | (+) | (+) | / |
|  | ITGβ4 IgG | (-) | (-) | / |
|  | ITGβ4 IgA | (+) | (+) | / |
|  |  |  |  |  |
| ELISAs using various RPs | |  |  |  |
|  | Dsg1 IgG (MBL) | (-) | (-) | (-) |
|  | Dsg3 IgG (MBL) | (-) | (-) | (-) |
|  | BP180 NC16a IgG (MBL) | (+) | (+) | (+) |
|  | BP180 NC16a IgG (in house) | (+) | (+) | (+) |
|  | LM332 IgG (in house) | (-) | (-) | (-) |
|  | ITGα6β4 ECD IgG (in house) | (-) | (+) | (-) |
|  | ITGβ4 ECD IgG (in house) | (-) | (+) | (-) |
|  | ITGβ4 ICD IgG (in house) | (-) | (-) | (-) |
|  | BP180 NC16a IgA (in house) | (+) | (+) | (+) |
|  | BP180 NC16a IgM (in house) | (+) | (+) | (-) |
|  | BP180 NC16a IgE (in house) | (-) | (-) | (-) |
|  | LM332 IgA (in house) | (-) | (+) | (-) |
|  |  |  |  |  |
| ELISAs using various peptides | |  |  |  |
|  | Dsg1 IgG | (+) | (+) | (+) |
|  | Dsg1 IgA | (-) | (-) | (-) |
|  | Dsg3 IgG | (-) | (-) | (-) |
|  | Dsg3 IgA | (-) | (+) | (-) |
|  | BP180 IgG | (+) | (+) | (+) |
|  | BP180 IgA | (+) | (+) | (+) |
|  | ITGβ4 IgG | (-) | (-) | (-) |
|  | ITGβ4 IgA | (+) | (+) | (-) |

IB, immunoblotting; Dsg, desmoglein; LM, laminin; RP, recombinant protein; ITG, integrin; ECD, extracellular domain; ICD, intracellular domain. “/”, not applicable. MBL, Medical and Biological Laboratories, CO., LTD, Tokyo Japan.
